# Vitamin C intake and osteoarthritis: findings of NHANES 2003–2018 and Mendelian randomization study

**DOI:** 10.3389/fnut.2024.1409578

**Published:** 2024-10-23

**Authors:** Hongjie Zhang, Xuan Jiang, Lei Bai, Jiahui Chen, Wei Luo, Jianxiong Ma, Xinlong Ma

**Affiliations:** ^1^Dehong People's Hospital, Kunming Medical University Affiliated Dehong Hospital, Mangshi, China; ^2^Tianjin University of Traditional Chinese Medicine, Tianjin, China; ^3^Tianjin Orthopedic Research Institute, Tianjin Hospital, Tianjin, China; ^4^Tianjin Medical University, Tianjin, China

**Keywords:** NHANES, vitamin C intake, osteoarthritis, Mendelian randomization, nutrition

## Abstract

**Background:**

The role of vitamin C in osteoarthritis (OA) is still a subject of debate. Our aim was to combine the National Health and Nutrition Examination Survey (NHANES) and MR studies to explore the relationship between vitamin C intake and OA.

**Methods:**

Clinical information on participants during NHANES 2003–2018 was collected and the relationship between vitamin C intake and OA risk was assessed using logistic regression modelling. In MR analyses, three methods were used to explore the causality of vitamin C intake with OA. Sensitivity analysis to verify the stability of the MR study.

**Results:**

The cross-sectional study included a total of 31,527 participants, categorizing them into low (<30.2 mg), medium (30.2–93.0 mg) and high (>93.0 mg) level groups based on their vitamin C intake levels. Logistic regression models showed that vitamin C intake was not associated with OA risk (*p* > 0.05). Inverse-variance weighted (IVW) method of MR study showed no causality between vitamin C intake and OA (OR = 0.993, 95% CI: 0.901 ~ 1.095, *p* = 0.882). Sensitivity analysis indicated that the MR study was reliable.

**Conclusion:**

Our cross-sectional and MR studies showed that vitamin C intake was not associated with OA risk. More researches are needed in the future to investigate the link between vitamin C and OA.

## Background

Osteoarthritis (OA) is a widespread and incapacitating disease that impacts joints, marked by the progressive breakdown of articular cartilage (1). Globally, it stands as a primary contributor to pain and functional impairment, with a pronounced impact on individuals, especially among the elderly (2). Unfortunately, as the population ages and obesity becomes more prevalent, the occurrence of OA is steadily increasing each year (3). The main treatment approach for OA is symptomatic management, aiming to alleviate symptoms such as swelling and pain, however, this is temporary (4). After the failure of symptomatic treatment, invasive methods such as joint replacement and intra-articular injections are employed. While effective, these approaches come with numerous side effects (5). Therefore, it is crucial to seek a treatment method for preventing and continually improving OA.

Reactive oxygen species (ROS) can induce the death of cartilage cells, constituting a crucial risk factor for age-related degenerative OA (6). Vitamin C, recognized for its potent antioxidant properties, serves as an effective scavenger of ROS (7). Considering this information, the antioxidant attributes of vitamin C could potentially offer a protective effect against OA. Yet, current researches on the relationship between vitamin C and OA remains controversial. An animal experiment conducted by Kurz et al. indicated that supplementing with vitamin C can prevent the development of OA (8). A cohort study revealed that vitamin C intake can alleviate bone lesions in patients with OA (9). However, a longitudinal study with a 4-year follow-up found no correlation between vitamin C and OA (10). Therefore, additional researches are needed to explore the connection between vitamin C and OA.

NHANES focuses on the nutritional and health situation of the U.S. population. The substantial sample size of NHANES performs a vital role in informing the development of public health policies and strategy for implementation (11). Mendelian randomization (MR) studies explore the causal relationship between exposure and outcome from a genetic dimension, greatly mitigating the effects of reverse causation and confounding factors (12, 13). To determine the link of vitamin C intake to OA, we are combining NHANES with Mendelian MR studies, aiming to provide theoretical support for the prevention and improvement of OA.

## Methods

### Participant selection

**Figure 1:**
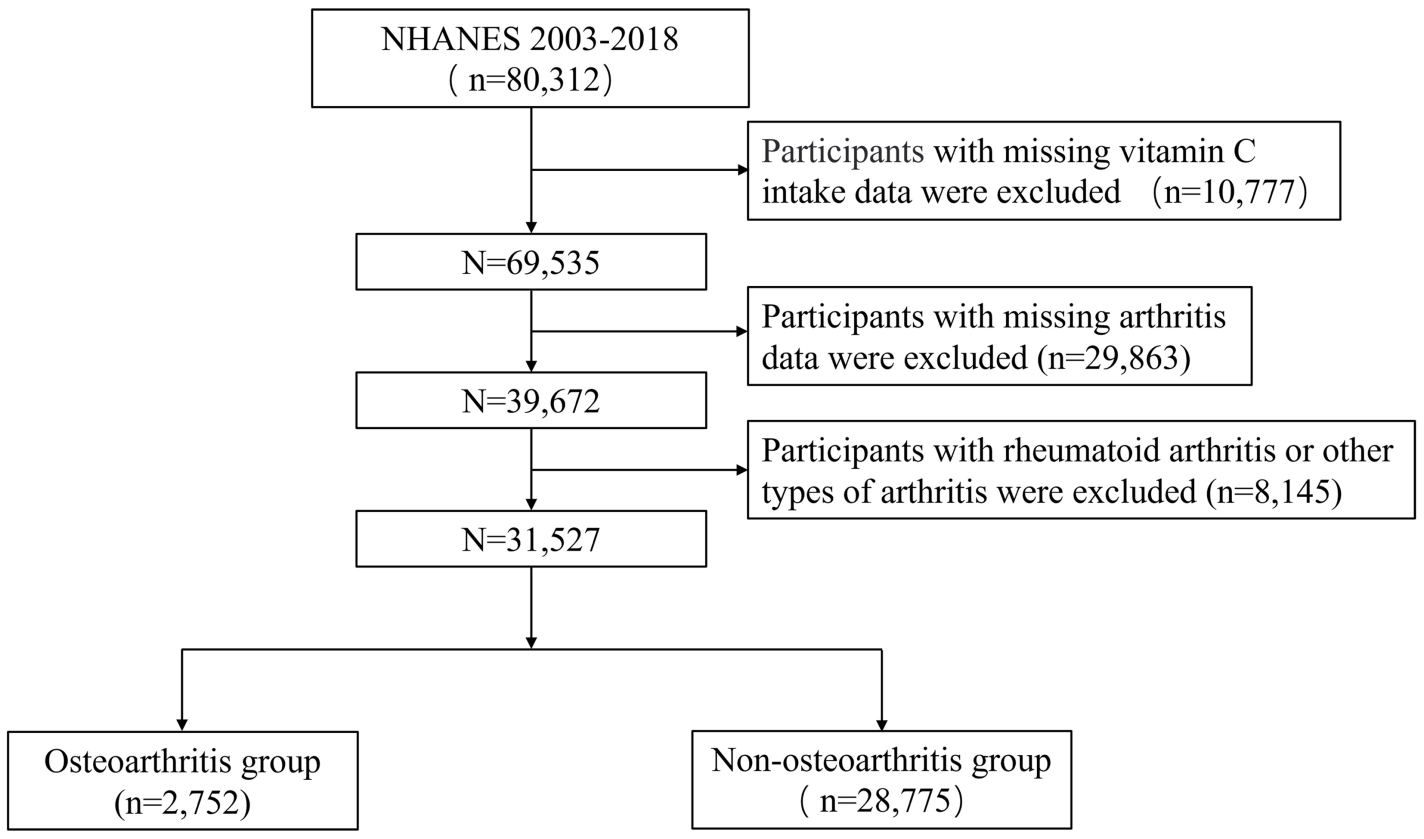
NHANES 2003–2018 participant’s selection flowchart.

We collected information from NHANES 2003–2018 and obtained a total of 80,312 individuals. Firstly, participants with incomplete vitamin C intake data were excluded. Secondly, individuals with incomplete arthritis information were excluded. Lastly, participants with rheumatoid arthritis or other types of arthritis were excluded (Figure 1).

### Variables in NHANES

Two questions on the questionnaire were used to determine whether participants had OA: “(1) Doctor ever said you had arthritis? (2) Which type of arthritis was it?” We obtained data on vitamin C intake through the Dietary Interview of Total Nutrient Intakes-First Day. Based on previous studies (14–16), the following variables are included in this study: gender, age, race, educational level, poverty-income ratio (PIR), marital status, smoking, drinking, physical activity, body mass index (BMI), diabetes, hypertension, hyperlipidemia, energy intake, protein intake and sugar intake. See Supplementary Table S1 for a full breakdown of these variables. All participants were equally divided in low, medium and high groups according to their vitamin C intake levels.

### Instrumental variables selection

**Figure 2:**
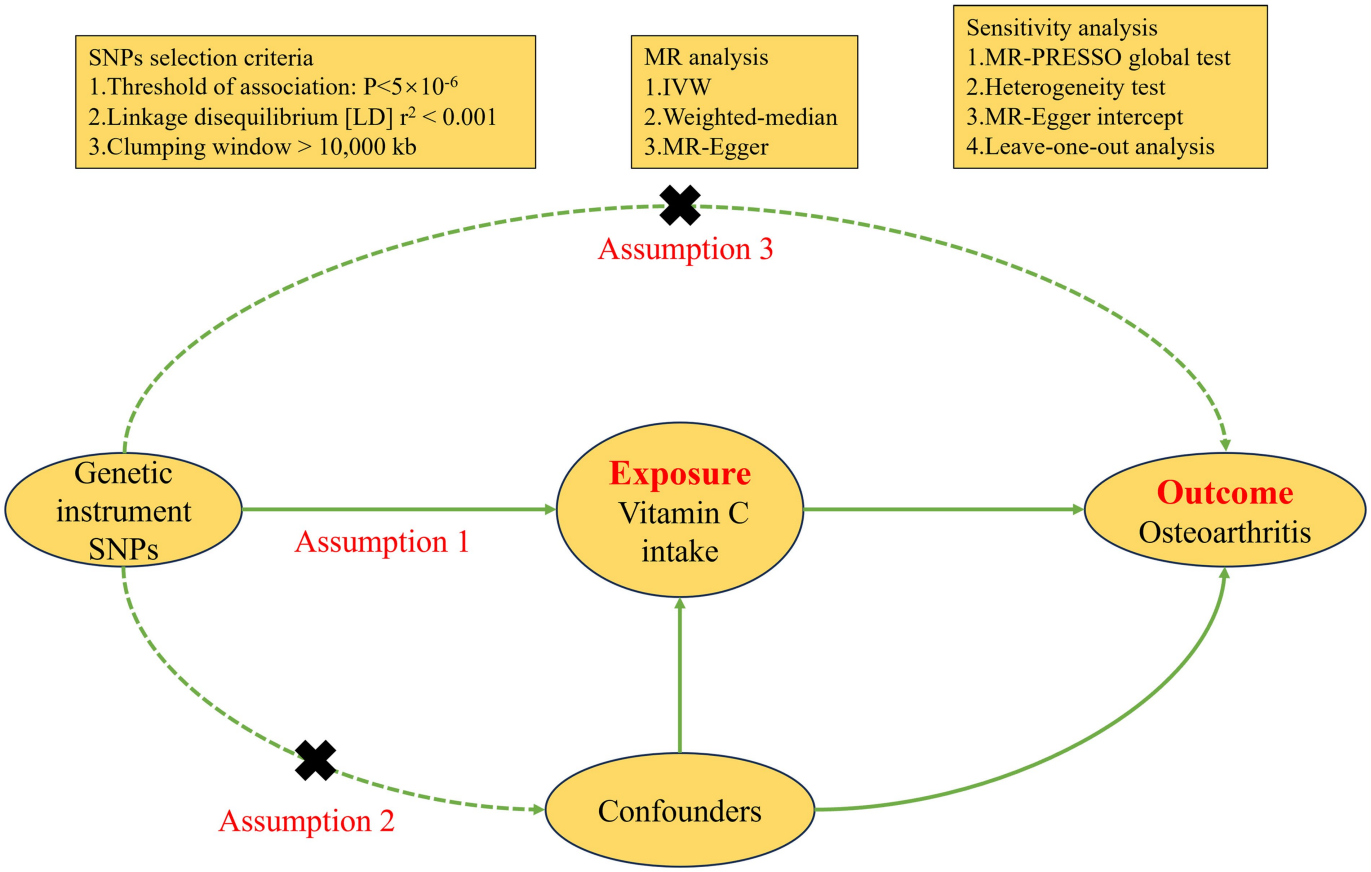
Diagram of the Mendelian randomization process.

Vitamin C intake served as the exposure variable in this study, we obtained instrumental variables (IVs) from a study on a European population in UK biobank. The Genome Wide Association Study (GWAS) summary data of vitamin C intake was extracted directly from the IEU open GWAS project (ID: ukb-b-15175). The study had the sample of 460,351 cases, of which 39,880 were patients and 420,471 were control. To test the effectiveness of IVs, we used the F statistic. *F* > 10 indicates that IVs are effective (17). The specific calculation method and explanation of F (18) can be found in Supplementary Table S2. The choice of IVs must meet the three main MR assumptions. First, Single Nucleotide Polymorphisms (SNPs) need to be strongly correlated with exposure (*p* < 5 × 10^−6^) and have F-statistics >10. Second, after determining linkage disequilibrium (LD) with *r*^2^ < 0.001 and clumping distance = 10,000 kb, independent SNPs were retained. Last, SNPs linked to the outcome and confounding variables were not included (*p* < 5 × 10^−6^). Based on these selection criteria, we obtained 25 SNPs associated with vitamin C intake (Supplementary Table S3). We searched for other phenotypes on PhenoScanner and did not find any SNPs associated with OA and confounding factors. Figure 2 illustrates the detailed process of MR.

### GWAS data summary of OA

We used OA as the outcome, which has GWAS data from a European population study by Dönertaş et al. (19). OA cases in this study were defined based on International Classification of Diseases (ICD)-10 coding (M15–M19, M47). This study contained a sample of 484,598 cases, including 39,515 patients. MR-PRESSO test did not find outlier SNPs. After harmonization, we found rs10000324 and rs12535840 as palindromic SNPs and excluded them, and finally 23 SNPs were included in the MR analysis. The impact which vitamin C intake has upon OA is shown at Supplementary Table S4.

### Statistical analysis

Graphing and analysis using R software (version 4.2.1). All analyses were conducted using survey weights provided by the NHANES to account for the complex, multistage probability sampling design. Categorical variables were expressed as weighted frequencies (%), and group comparisons were conducted by the weighted chi-square test. For continuous variables, data were presented as mean and standard error (SE), group comparisons were conducted using weighted linear regression models. We developed three multivariate logistic regression models for evaluating the link of vitamin C intake to OA. Crude model: not adjusted; model 1: adjusted for age, gender, race, educational level, PIR and marital status; model 2: adjusted for smoking, drinking, physical activity, BMI, diabetes, hypertension, hyperlipidemia, energy intake, protein intake and sugar intake based on model 1.

Inverse-variance weighted (IVW) served as the main method of MR analysis, with MR Egger and weighted median as supplements (20). The findings from the MR analysis were presented by odds ratios (ORs), representing the increased hazard of OA per unit rise in vitamin C intake. MR-Egger intercepts were applied for testing horizontal pleiotropy in SNPs, with an intercept close to 0 indicating the absence of pleiotropy (*p* > 0.05) (21). Heterogeneity of SNPs was checked by Cochran’s Q test, and no heterogeneity was required to satisfy *p* > 0.05 and *I^2^* < 25%. Reliability of MR analysis assessed by Funnel plot symmetry and Leave-one-out. The R packages used in MR analysis are “MRPRESSO” and “TwoSampleMR.” *p* < 0.05 was considered statistically significant.

## Results

### Clinical features

The research included 31,527 individuals, the average age of whom was 44.4 ± 0.2 years; 50.1% of the participants were male and 49.9% were female. All participants were divided into tertiles based on their vitamin C intake levels: low (<30.2 mg), medium (30.2–93.0 mg) and high (>93.0 mg) groups. In the entire study, 7.7% of individuals had OA. The incidence of OA in the three groups was 7.2, 7.8, and 8.3%, respectively; the difference was not statistically significant (*p* > 0.05). As shown in Table 1, race, gender, age, educational level, PIR, marital status, BMI, smoking, drinking, physical activity, energy, protein, and sugar intakes (all *p* < 0.05) were statistically significant compared to the three groups. Compared to the low group, participants in the high group were older, more likely to be male, Mexican American, accompanied by a partner, had higher education levels, were wealthier, exercised more, had higher energy, protein, and sugar intake, and had less smoking and drinking history, as well as lower body weight (all *p* < 0.05).

**Table 1:** Weighted clinical features of the participants grouped by tertile of the vitamin C intake.

Variables	Total (*n* = 31,527)	<30.2 mg (*n* = 10,505)	30.2–93.0 mg (*n* = 10,513)	>93.0 mg (*n* = 10,509)	*p*
Gender, *n* (%)					<0.001
Male	15,817 (50.1)	5,212 (48.8)	5,104 (48.3)	5,501 (53.0)	
Female	15,710 (49.9)	5,293 (51.2)	5,409 (51.7)	5,008 (47.0)	
Age (years), mean (SE)	44.4 (0.2)	42.8 (0.2)	45.3 (0.3)	45.0 (0.3)	<0.001
Race, *n* (%)					<0.001
Mexican American	5,451 (9.3)	1,665 (8.4)	1,862 (9.1)	1,924 (10.3)	
Other Hispanic	2,828 (5.6)	873 (5.3)	905 (5.3)	1,050 (6.5)	
Non-Hispanic White	13,201 (66.2)	4,685 (67.9)	4,513 (67.9)	4,003 (62.7)	
Non-Hispanic Black	6,679 (11.3)	2,350 (11.8)	2,014 (9.9)	2,315 (12.4)	
Other races	3,368 (7.6)	932 (6.6)	1,219 (7.8)	1,217 (8.1)	
Educational level, *n* (%)					<0.001
Under high school	7,527 (15.3)	2,750 (17.5)	2,439 (14.0)	2,338 (14.4)	
High school or equivalent	7,227 (23.4)	2,714 (27.4)	2,358 (22.6)	2,155 (19.9)	
College or above	16,773 (61.3)	5,041 (55.1)	5,716 (63.3)	6,016 (65.7)	
PIR, *n* (%)					<0.001
<2	13,514 (31.6)	5,068 (36.3)	4,205 (28.5)	4,241 (29.9)	
≥2	15,521 (62.0)	4,645 (57.5)	5,479 (65.4)	5,397 (63.3)	
Not record	2,492 (6.4)	792 (6.2)	829 (6.1)	871 (6.8)	
Marital status, *n* (%)					<0.001
Coupled	19,134 (63.8)	5,974 (59.6)	6,562 (66.1)	6,598 (65.6)	
Not coupled	12,393 (36.2)	4,531 (40.4)	3,951 (33.9)	3,911 (34.4)	
Smoking, *n* (%)					<0.001
Never	17,975 (56.6)	5,363 (50.5)	6,176 (59.0)	6,436 (60.6)	
Former	7,003 (22.7)	2,152 (20.8)	2,420 (23.1)	2,431 (24.4)	
Current	6,549 (20.7)	2,990 (28.7)	1,917 (17.9)	1,642 (15.0)	
Drinking, *n* (%)					<0.001
Never	11,170 (29.0)	3,697 (29.3)	3,758 (28.8)	3,715 (29.0)	
Mild	9,583 (33.3)	2,800 (28.6)	3,334 (34.9)	3,449 (36.6)	
Moderate	4,577 (16.6)	1,573 (16.8)	1,495 (16.5)	1,509 (16.4)	
Heavy	6,197 (21.1)	2,435 (25.3)	1,926 (19.8)	1,836 (18.0)	
Physical activity, *n* (%)					<0.001
Vigorous physical activity	11,697 (42.4)	3,721 (39.9)	3,774 (41.2)	4,202 (46.3)	
No vigorous activity	19,830 (57.6)	6,784 (60.1)	6,739 (58.8)	6,307 (53.7)	
BMI (kg/m^2^), mean (SE)	28.5 (0.1)	28.9 (0.1)	28.6 (0.1)	28.0 (0.1)	<0.001
Diabetes, *n* (%)					0.090
Yes	4,668 (10.8)	1,593 (11.0)	1,640 (11.3)	1,435 (10.0)	
No	26,525 (88.2)	8,801 (87.9)	8,748 (87.7)	8,976 (89.1)	
Borderline	334 (1.0)	111 (1.1)	125 (1.0)	98 (0.9)	
Hypertension, *n* (%)					0.372
Yes	11,144 (31.0)	3,774 (31.0)	3,789 (31.6)	3,581 (30.3)	
No	20,383 (69.0)	6,731 (69.0)	6,724 (68.4)	6,928 (69.7)	
Hyperlipidemia, *n* (%)					0.351
Yes	18,005 (56.6)	5,917 (55.9)	6,099 (57.1)	5,989 (56.7)	
No	13,185 (42.5)	4,450 (43.0)	4,317 (42.1)	4,418 (42.4)	
Not record	337 (0.9)	138 (1.1)	97 (0.8)	102 (0.9)	
Energy intake (kcal), mean (SE)	2224.3 (7.9)	1986.5 (11.5)	2236.4 (12.5)	2464.2 (15.3)	<0.001
Protein intake (g), mean (SE)	85.0 (0.4)	74.4 (0.5)	86.7 (0.6)	94.4 (0.6)	<0.001
Sugar intake (g), mean (SE)	117.7 (0.7)	102.3 (1.1)	111.4 (1.1)	141.0 (1.2)	<0.001
Osteoarthritis, *n* (%)					0.073
Yes	2,752 (7.7)	875 (7.2)	944 (7.8)	933 (8.3)	
No	28,775 (92.3)	9,630 (92.8)	9,569 (92.2)	9,576 (91.7)	

CI, confidence interval; PIR, poverty-income ratio; SE, standard error, BMI, body mass index.

### Correlation of vitamin C intake with OA

Weighted Logistic regression analysis with vitamin C intake level as a continuous variable showed no correlation of vitamin C intake with OA (OR = 0.991, *p* = 0.710). After adjusting the variables, both model 1 and 2 still do not find a correlation between them (OR = 1.013, 1.011; *p* = 0.676, 0.715). After categorizing the vitamin C intake level, none of the three models detected a link of vitamin C intake to OA (*p* > 0.05). Details are given in Table 2.

**Table 2:** Weighted logistic regression analysis of the correlation between vitamin C intake and OA.

	Crude model		Model 1		Model 2	
	OR (95% CI)	*p*	OR (95% CI)	*p*	OR (95% CI)	*p*
Vitamin C intake (100 mg)	0.991 (0.943, 1.041)	0.710	1.013 (0.954, 1.075)	0.676	1.011 (0.953, 1.073)	0.715
Vitamin C intake categories						
<30.2 mg	Reference		Reference		Reference	
30.2–93.0 mg	1.090 (0.968, 1.227)	0.155	0.935 (0.819, 1.066)	0.310	0.999 (0.869, 1.148)	0.988
>93.0 mg	1.162 (1.013, 1.332)	0.032	1.046 (0.902, 1.213)	0.546	1.103 (0.945, 1.289)	0.213

Crude model, not adjusted; Model 1, adjusted for age, gender, race, educational level, PIR and marital status; Model 2, additionally adjusted for smoking, drinking, physical activity, BMI, diabetes, hypertension, hyperlipidemia, energy intake, protein intake and sugar intake. OR, odds ratio; CI, confidence interval; PIR, poverty-income ratio; BMI, body mass index.

### Causality and MR sensitivity analysis of vitamin C intake and OA

**Figure 3:**
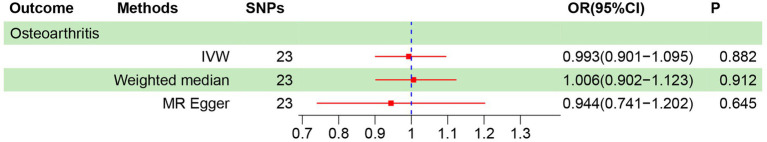
MR results concerning vitamin C intake and OA.

There are 23 SNPs in the MR analysis that all had *F* > 10 (Supplementary Table S3). As depicted in Figure 3, IVW, weighted median and MR-Egger models revealed no causality between vitamin C intake and OA (OR = 0.993, 1.006, 0.944; all *p* > 0.05). We also plotted the forest plot for each SNP assessing the causality of vitamin C intake and OA (Supplementary Figure S1). Mild heterogeneity existed among SNPs (*p* = 0.066, *I^2^* = 32.8%) (Table 3). MR-Egger intercept analysis showed no horizontal pleiotropy (intercept = 2.30E-04, *p* = 0.659) (Table 3; Supplementary Figure S2). MR-PRESSO test revealed no outlier SNPs (*p* = 0.075) (Table 3). No single SNP highly influences the overall effect in leave-one-out analysis (Supplementary Figure S3). In addition, the symmetry of funnel plot was better, showing the pleiotropy did not exist (Supplementary Figure S4). The sensitivity analysis results above suggest the reliability on MR results.

**Table 3:** Sensitivity analysis of MR.

Outcome	MR-PRESSO			MR-Egger			Cochran Q test		
	Casual estimate	SD	Global test *p*	intercept	SE	*p*	Q value	*p*	*I^2^*
Osteoarthritis	−0.007	0.050	0.075	2.30E-04	5.14E-04	0.659	32.738	0.066	32.8%

## Discussion

In NHANES, logistic regression analysis revealed no link of vitamin C intake to OA. Subsequent MR analyses also confirmed the absence of causality between the two.

OA mainly influences weight-bearing joints, with hands and knees being the most common. The main pathological manifestations include synovial inflammation and subchondral bone damage in habitual weight-bearing areas. In severe cases, osteophytes may form, leading to pain, swelling, limited mobility, crepitus, and a grinding sensation in the affected joints. The joint cavity may exhibit varying degrees of exudative inflammation, while systemic symptoms are generally less common (22–24). In OA, oxidative stress resulting from sustained ROS production can promote cartilage degradation through signaling pathways such as PI3K/AKT, leading to cartilage degeneration (25). Vitamin C can play a beneficial role in preventing cartilage damage and slow down the progression of OA by inhibiting lipid oxidation through its antioxidative capabilities (26). Antioxidant levels in joint fluid are often significantly lower in patients with severe OA compared to normal subjects (27). However, the findings from multiple investigations regarding the link of vitamin C to OA are inconsistent. Peregoy et al.’s clinical study, which included a total of 1,023 patients aged 40 and above, revealed that supplementing with vitamin C could reduce the incidence risk of OA by 11% after a 20-year follow-up (28). McAlindon et al. conducted a study with 640 participants to investigate the potential of antioxidant intake in lowering the risk of OA. The findings indicated a significant decrease in the advancement of OA and cartilage loss with elevated levels of vitamin C supplementation (29). However, other studies have concluded no link was found for vitamin C and OA, or that vitamin C can increase the OA risk. Veen et al.’s study involved 43,865 participants, with 5,976 cases in the case group, followed all participants for 19 years. After adjusting for confounding factors, the study found no link of vitamin C supplementation to OA (30). The research by Joseph et al. that included 1,785 participants also failed to find a link between vitamin C intake and OA (10). A total of 4,685 participants were included in Li et al. ‘s study that explored the connection of supplementation with antioxidants and OA, and found that vitamin C increased OA risk (31).

In the cross-sectional study from NHANES, we first divided participants into OA and non-OA groups, and logistic regression analyses after adjusting for confounders confirmed that no correlation was found among vitamin C intake and OA. We then transformed vitamin C intake level into categorical variables and still found no association. The results of our observational study are consistent with those of Veen et al. To further confirm our findings, MR analyses were performed. We used 23 SNPs strongly associated with vitamin C intake as IVs to perform MR analysis, and found that there was no causality between vitamin C intake and OA, and sensitivity analyses demonstrated that our MR analyses were reliable. The MR analyses validated the results obtained from the observational study at the genetic level. We speculate that the probable reason is that the etiology of OA is complex and many of the pathogenic mechanisms are still unclear (32). Although the vitamin C has antioxidant function, it cannot exert a decisive function on the pathogenesis of OA, and therefore its association with OA is not obvious. Additionally, individuals have varying requirements for vitamin C, making it challenging to determine the appropriate dosage when supplementing. Excessive intake of vitamin C may have negative effects on the body (33, 34). The innovation of our study is to combine NHANES and MR studies to explore the connection of vitamin C intake and OA in epidemiological and genetic perspectives, avoiding the influence of many confounding factors on the results and making them more scientific and reliable. The consistent results of the two methods also make the conclusions more convincing. Nevertheless, our study has some limitations. First, different ethnicities included in cross-sectional and MR studies may bias results. As our study only included European and US populations, our conclusions have yet to be validated in other populations. Second, we did not explore the association of vitamin C deficiency with the risk of OA. Last, we did not explore the specific underlying mechanisms by which the antioxidant effects of vitamin C may affect OA, and future studies are needed to explore them further.

## Conclusion

In summary, in the study from NHANES, logistic regression analyses did not find an association between vitamin C intake and the risk of OA. Further MR analyses also showed no causality between vitamin C intake and OA, and the conclusions were consistent between the two research methods. More studies are needed to explore the relationship between vitamin C and OA in the future.

## Data Availability

The original contributions presented in the study are included in the article/Supplementary material, further inquiries can be directed to the corresponding authors.
