# Supplementary material for: Vitamin C intake and osteoarthritis: findings of NHANES 2003–2018 and Mendelian randomization study
Source: Front Nutr. 2024 Oct 23;11:1409578. doi: 10.3389/fnut.2024.1409578 (PMC11537885; doi:10.3389/fnut.2024.1409578)
Supplement: Supplementary file 1 [file Table_1.docx]

**Supplementary Table 1: Description of covariates.**

| Covariates | Description in NHANES |
| --- | --- |
| Age | Continuous variable |
| Gender | Male and Female |
| Race | Mexican American, Non-Hispanic Black, Non-Hispanic White, Other Race |
| Educational level | Under high school, High school or equivalent, College or above |
| Marital status | Coupled: Married/Living with partner; Not coupled: Widowed/ Divorced/ Separated/ Never married |
| PIR | Poor: <2; Not Poor:>=2 |
| BMI | Continuous variable |
| Smoking | Smoking status was grouped into never smoker (defined as <100 cigarettes in a lifetime), current smoker (defined as ≥100 cigarettes in a lifetime), and former smoker (defined as ≥100 cigarettes and had quit smoking) |
| Drinking | heavy drinking (≥4 drinks/day for men, ≥3 drinks/day for women, or ≥5 days of drinking in a month),  moderate drinking (≥3 drinks/day for men, ≥2 drinks/day for women, or ≥2 days of drinking in a month),  mild drinking (≤2 drinks/day for men, ≤1 drink/day for women, and ≥12 drinks in a year),  and never-drinking (total number of drinks in a year <12, and dietary alcohol content of 0%) |
| Physical activity | Vigorous physical activity: physical activities including exercise, sports, and physically active hobbies that {you/SP} may have done in {your/his/her} leisure time or at school over the past 30 days (NHANES 2003-2006); Vigorous recreational activities or Vigorous work activity (NHANES2007-2018) |
| Diabetes | Diabetes was defined as a history of previous diabetes, HbA1c level ≥6.5%, or fasting blood glucose level ≥126 mg/dL |
| Hypertension | The diagnostic criteria consist of self-reported hypertension history, the utilization of antihypertensive medication, a systolic blood pressure (SBP) ≥ 140mmHg, or a diastolic blood pressure (DBP) ≥ 90mmHg |
| Hyperlipidemia | TG levels ≥ 150 mg/ dL or hypercholesterolemia.  Hypercholesterolemia:  a. TC levels ≥ 200 mg/dL;  b. or LDL-C levels ≥ 130 mg/dL;  c. or HDL-C levels < 40 mg/dL for males and < 50 mg/dL for females. |
| Energy intake | Continuous variable (Dietary Interview of Total Nutrient Intakes-First Day) |
| Protein intake | Continuous variable (Dietary Interview of Total Nutrient Intakes-First Day) |
| Sugar intake | Continuous variable (Dietary Interview of Total Nutrient Intakes-First Day) |

**PIR, Ratio of family income to poverty;** **BMI,** **body mass index.**

**Supplementary Table 2: The specific calculation method and explanation of F**

| Index | Formulas/descriptions |
| --- | --- |
| F | R^2^(N-2)/(1-R^2^) |
| R^2^ | (2×β^2^×eaf×(1-eaf)) / [(2×β^2^×eaf×(1-eaf))+ (2×N×SE^2^×eaf×(1-eaf))] |
| N | The sample size in the exposure GWAS |
| eaf | The effect allele frequency of the IVs |
| β | The beta value of the IVs |
| SE | Standard error of β |

**Supplementary Table 3: Features of 25 SNPs used as instrumental variables for exposure (vitamin C intake).**

|  | SNP | EA | OA | EAF | effect | SE | N | p-value | F-Statistic |
| --- | --- | --- | --- | --- | --- | --- | --- | --- | --- |
| 1 | rs113962806 | T | C | 0.0406 | 0.0070 | 0.0015 | 460,351 | 3.50E-06 | 21.50 |
| 2 | rs72662628 | C | T | 0.0146 | 0.0112 | 0.0024 | 460,351 | 4.50E-06 | 21.03 |
| 3 | rs59538793 | T | C | 0.3037 | 0.0031 | 0.0006 | 460,351 | 1.50E-06 | 23.14 |
| 4 | rs185845702 | C | A | 0.0103 | -0.0141 | 0.0030 | 460,351 | 3.30E-06 | 21.61 |
| 5 | rs10910087 | G | A | 0.7092 | 0.0030 | 0.0007 | 460,351 | 4.40E-06 | 21.10 |
| 6 | rs116025296 | A | G | 0.0160 | 0.0117 | 0.0025 | 460,351 | 2.50E-06 | 22.20 |
| 7 | rs74634742 | T | G | 0.0484 | 0.0064 | 0.0014 | 460,351 | 3.60E-06 | 21.48 |
| 8 | rs62144051 | G | A | 0.0936 | -0.0047 | 0.0010 | 460,351 | 3.40E-06 | 21.56 |
| 9 | rs67914003 | T | C | 0.0514 | -0.0066 | 0.0014 | 460,351 | 1.10E-06 | 23.70 |
| 10 | rs6796394 | C | G | 0.7798 | 0.0033 | 0.0007 | 460,351 | 4.10E-06 | 21.24 |
| 11 | rs72993633 | G | A | 0.0500 | 0.0066 | 0.0013 | 460,351 | 8.50E-07 | 24.24 |
| 12 | rs10000324 | T | A | 0.4255 | -0.0028 | 0.0006 | 460,351 | 1.90E-06 | 22.67 |
| 13 | rs35751866 | A | C | 0.2513 | 0.0032 | 0.0007 | 460,351 | 2.80E-06 | 21.98 |
| 14 | rs74797698 | A | T | 0.0303 | 0.0080 | 0.0017 | 460,351 | 3.00E-06 | 21.80 |
| 15 | rs2523735 | G | C | 0.1482 | -0.0042 | 0.0008 | 460,351 | 4.00E-07 | 25.70 |
| 16 | rs12535840 | G | C | 0.5829 | -0.0028 | 0.0006 | 460,351 | 3.30E-06 | 21.62 |
| 17 | rs904017 | A | C | 0.2933 | 0.0033 | 0.0006 | 460,351 | 4.30E-07 | 25.57 |
| 18 | rs11636071 | G | A | 0.7244 | 0.0030 | 0.0007 | 460,351 | 4.00E-06 | 21.29 |
| 19 | rs8063219 | G | T | 0.3811 | -0.0030 | 0.0006 | 460,351 | 9.50E-07 | 24.02 |
| 20 | rs4795397 | G | A | 0.4811 | -0.0032 | 0.0006 | 460,351 | 4.50E-08 | 29.93 |
| 21 | rs12960708 | A | G | 0.7560 | -0.0034 | 0.0007 | 460,351 | 7.00E-07 | 24.62 |
| 22 | rs307119 | G | A | 0.5889 | -0.0029 | 0.0006 | 460,351 | 1.60E-06 | 23.04 |
| 23 | rs79016212 | C | T | 0.0203 | 0.0101 | 0.0022 | 460,351 | 3.00E-06 | 21.81 |
| 24 | rs117654917 | A | G | 0.0154 | 0.0118 | 0.0026 | 460,351 | 3.90E-06 | 21.32 |
| 25 | rs78684519 | G | A | 0.0318 | 0.0070 | 0.0017 | 460,351 | 4.30E-06 | 21.15 |

SNPs, single nucleotide polymorphisms; EA, effect_allele; OA, other_allele; EAF, effect allele frequency; SE, standard error.

**Supplementary Table 4: Genetic association estimates for the effect of vitamin C intake on Osteoarthritis.**

**exposure= vitamin C intake;** **outcome= Osteoarthritis**

|  | SNP | beta.exposure | se.exposure | beta.outcome | se.outcome |
| --- | --- | --- | --- | --- | --- |
| 1 | rs10910087 | 0.0030 | 0.0007 | -1.98E-05 | 0.0006 |
| 2 | rs113962806 | 0.0070 | 0.0015 | -0.0014 | 0.0014 |
| 3 | rs116025296 | 0.0117 | 0.0025 | -0.0047 | 0.0024 |
| 4 | rs11636071 | 0.0030 | 0.0007 | 3.79E-05 | 0.0006 |
| 5 | rs117654917 | 0.0118 | 0.0026 | -0.0004 | 0.0024 |
| 6 | rs12960708 | -0.0034 | 0.0007 | 0.0012 | 0.0006 |
| 7 | rs185845702 | -0.0141 | 0.0030 | -0.0011 | 0.0029 |
| 8 | rs2523735 | -0.0042 | 0.0008 | 0.0025 | 0.0008 |
| 9 | rs307119 | -0.0029 | 0.0006 | -1.99E-05 | 0.0006 |
| 10 | rs35751866 | 0.0032 | 0.0007 | 3.73E-05 | 0.0006 |
| 11 | rs4795397 | -0.0032 | 0.0006 | 1.85E-05 | 0.0005 |
| 12 | rs59538793 | 0.0031 | 0.0006 | 0.0015 | 0.0006 |
| 13 | rs62144051 | -0.0047 | 0.0010 | -0.0005 | 0.0010 |
| 14 | rs67914003 | -0.0066 | 0.0014 | -9.89E-05 | 0.0013 |
| 15 | rs6796394 | 0.0033 | 0.0007 | 0.0005 | 0.0007 |
| 16 | rs72662628 | 0.0112 | 0.0024 | 3.78E-05 | 0.0023 |
| 17 | rs72993633 | 0.0066 | 0.0013 | -0.0003 | 0.0013 |
| 18 | rs74634742 | 0.0064 | 0.0014 | 0.0005 | 0.0013 |
| 19 | rs74797698 | 0.0080 | 0.0017 | 0.0034 | 0.0016 |
| 20 | rs78684519 | -0.0077 | 0.0017 | -0.0011 | 0.0016 |
| 21 | rs79016212 | 0.0101 | 0.0022 | -0.0011 | 0.0021 |
| 22 | rs8063219 | -0.0030 | 0.0006 | 0.0002 | 0.0006 |
| 23 | rs904017 | 0.0033 | 0.0006 | 0.0007 | 0.0006 |

SNPs, single nucleotide polymorphisms; se, standard error.
