# Supplementary material for: Vitamin C intake and osteoarthritis: findings of NHANES 2003–2018 and Mendelian randomization study
Source: Front Nutr. 2024 Oct 23;11:1409578. doi: 10.3389/fnut.2024.1409578 (PMC11537885; doi:10.3389/fnut.2024.1409578)

Supplementary Figure 1. Forest plot of SNPs associated with Vitamin C intake and Osteoarthritis. MR, mendelian randomization; SNPs, single nucleotide polymorphisms.

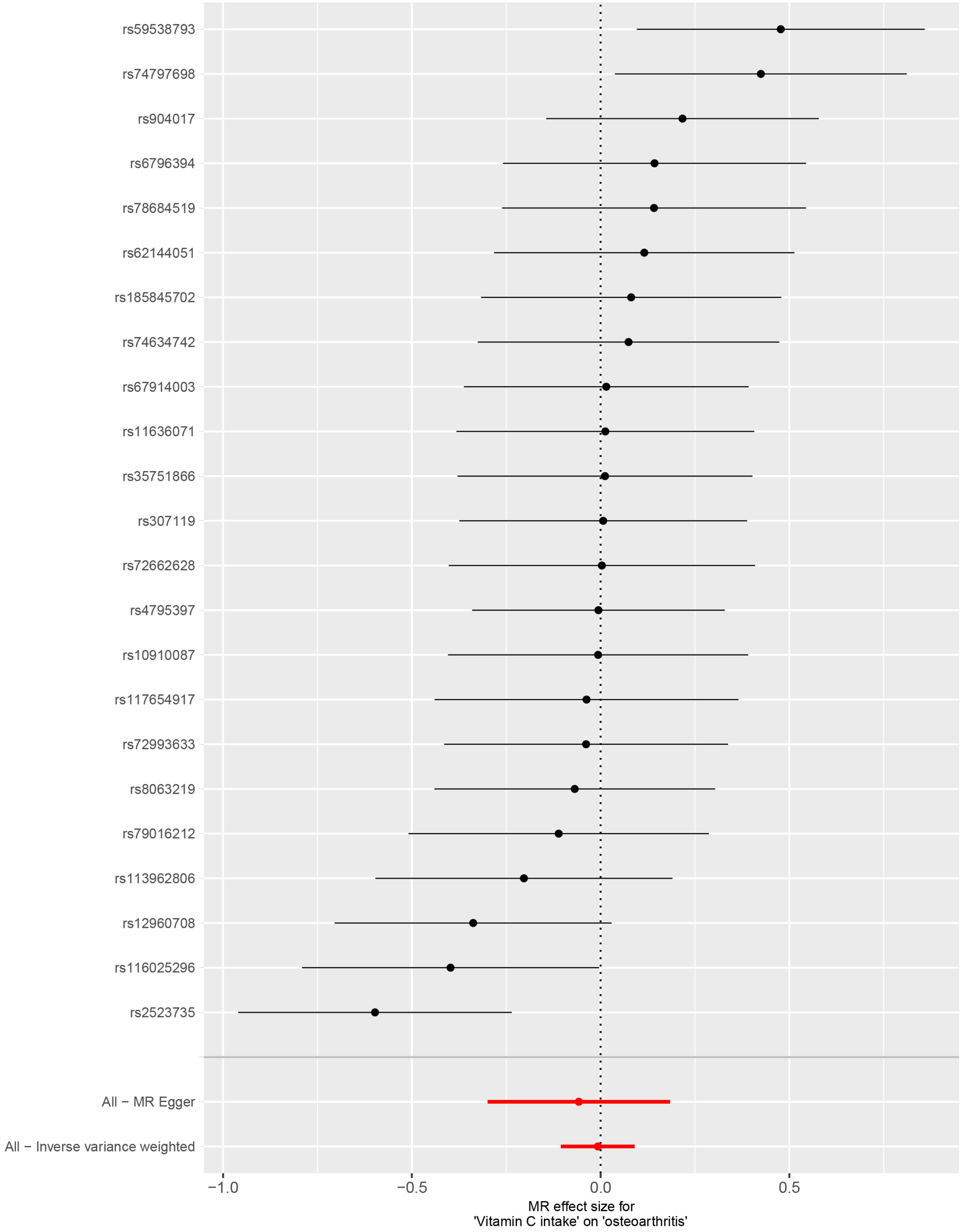

Supplementary Figure 2. Scatter plot of SNPs associated with Vitamin C intake and Osteoarthritis. MR, mendelian randomization; SNPs, single nucleotide polymorphisms.

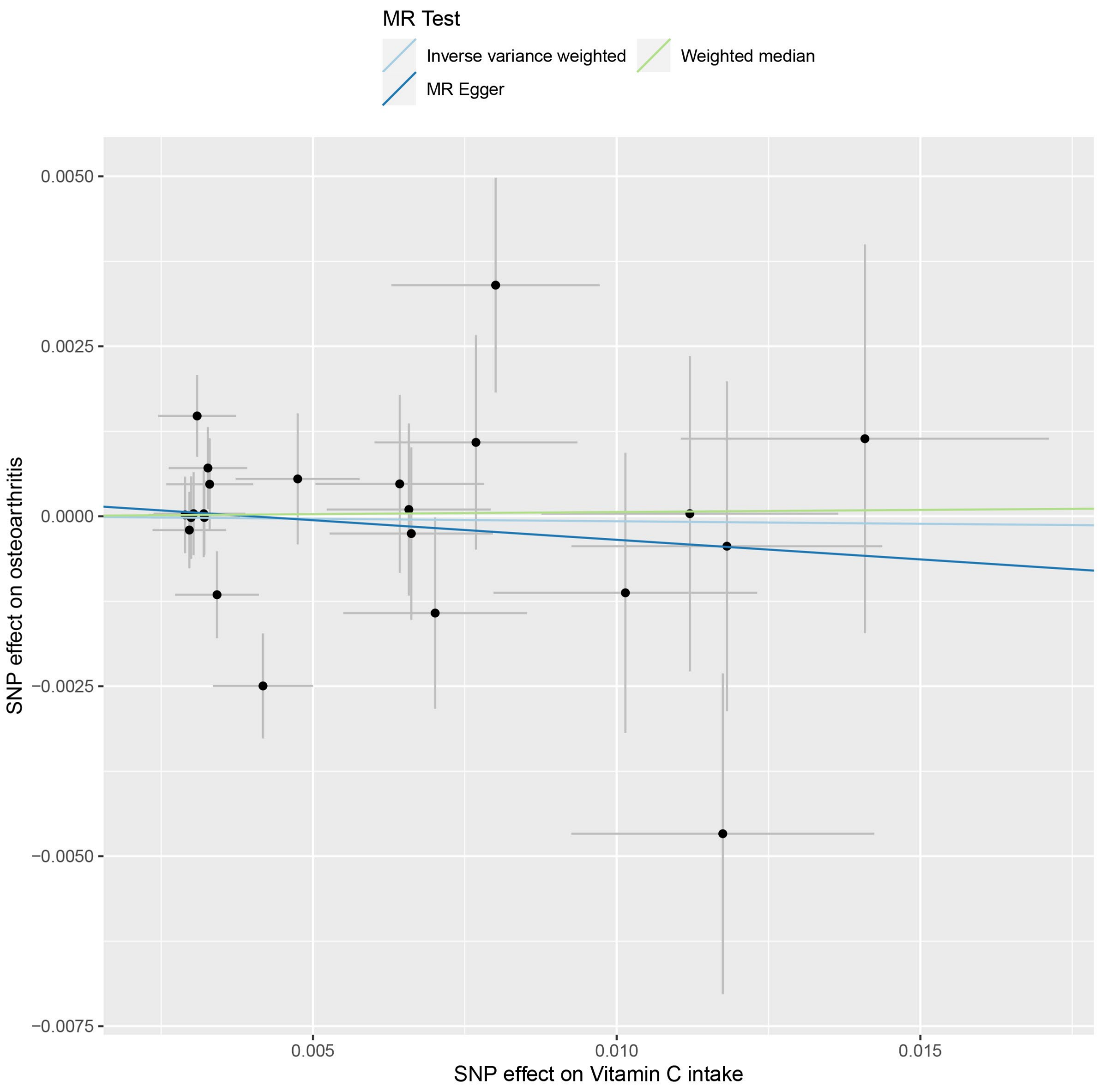

Supplementary Figure 3. Leave-one-out of SNPs associated with Vitamin C intake and Osteoarthritis. MR, mendelian randomization; SNPs, single nucleotide polymorphisms.

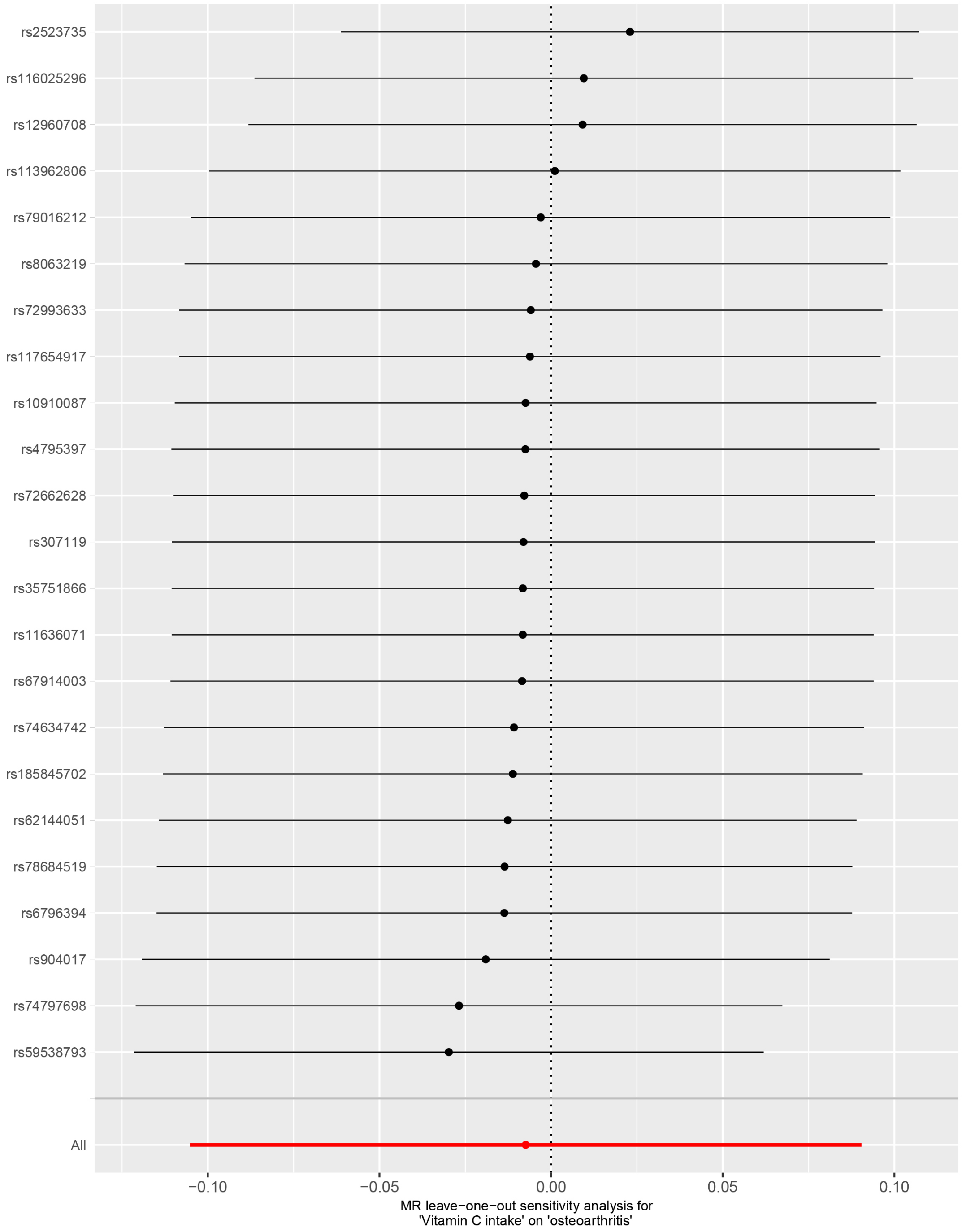

Supplementary Figure 4. Funnel plot of SNPs associated with Vitamin C intake and Osteoarthritis. MR, mendelian randomization; SNPs, single nucleotide polymorphisms.

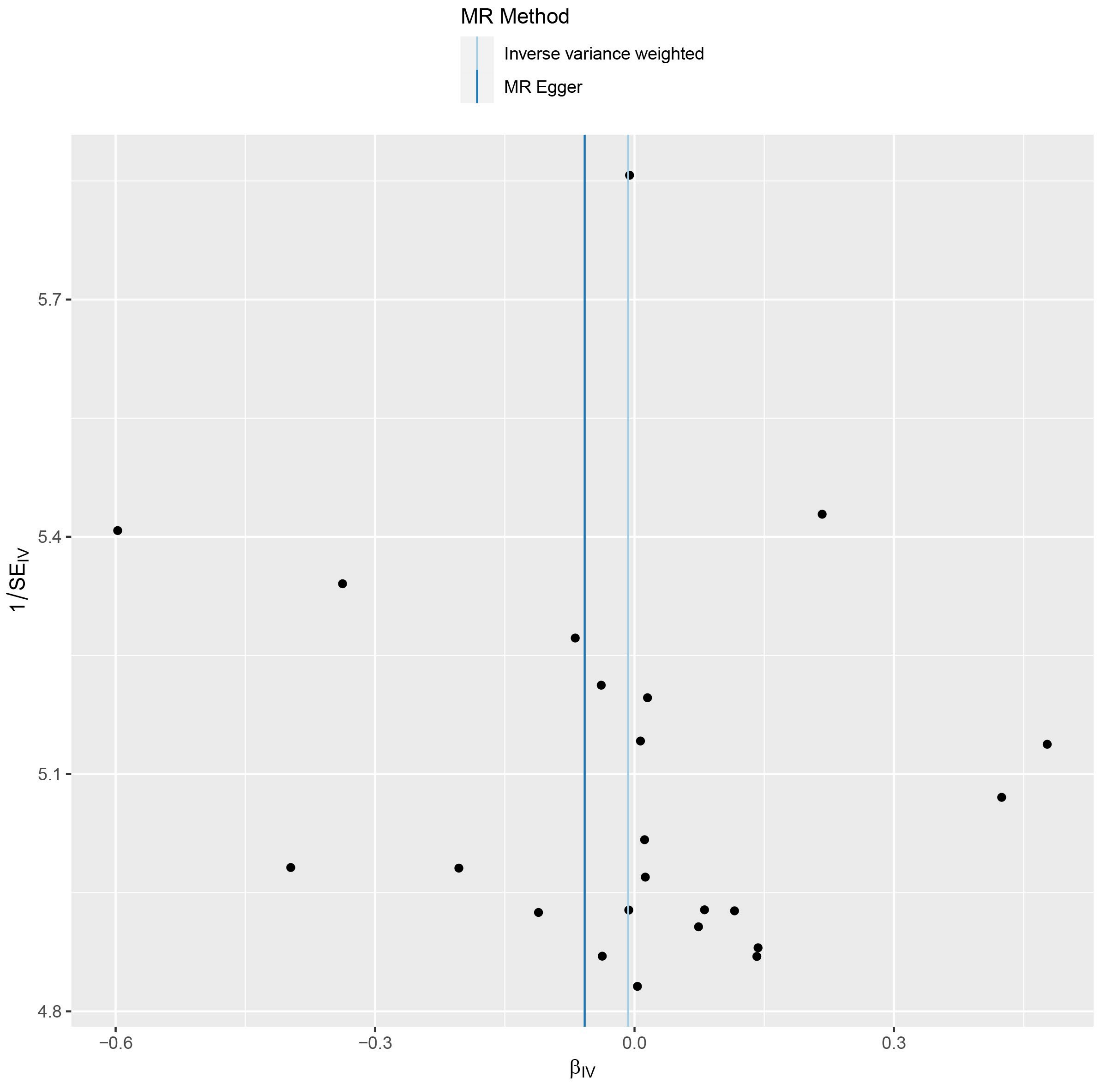

Supplement: Supplementary file 2 [file Image_1.pdf]
